# ECM1 promotes migration and invasion of hepatocellular carcinoma by inducing epithelial-mesenchymal transition

**DOI:** 10.1186/s12957-016-0952-z

**Published:** 2016-07-27

**Authors:** Hao Chen, Weidong Jia, Jiansheng Li

**Affiliations:** Department of Hepatic Surgery, Affiliated Provincial Hospital, Anhui Medical University, Anhui Province Key Laboratory of Hepatopancreatobiliary Surgery, 17 Lujiang Road, Hefei, 230001 Anhui Province People’s Republic of China

**Keywords:** Extracellular matrix protein 1, Epithelia-mesenchymal transition, Hepatocellular carcinoma, Migration, Invasion

## Abstract

**Background:**

Extracellular matrix protein 1 (ECM1) is a glycoprotein involved in many biologic processes. To determine the expression of ECM1 in hepatocellular carcinoma (HCC), and to study the role of ECM1 in inducing epithelia-mesenchymal transition (EMT) to analyze the effect of ECM1 on the migration and invasion of HCC cells.

**Methods:**

The expression of ECM1 in HCC specimens was examined by immunohistochemistry staining, and the correlations were analyzed between the expression of ECM1 and the clinicopathological data. The ECM1 expression plasmid was transfected into Bel-7402 cells to induce exogenous overexpression of ECM1 protein. The changes of the expression of ECM1, EMT-related protein (E-cadherin, Vimentin), in Bel-7402 cells were detected by Western blot after transfection of ECM1; the wound healing and invasion assay in vitro were used to determine the role of ECM1 gene transfection on the ability of migration and invasive potential of Bel-7402 cells.

**Results:**

Immumohistochemistry staining method displayed the ECM1 expression was positively associated with vascular invasion, TNM stage, and poor prognosis. A significant positive correlation was found between the expressions of ECM1 and Vimentin. After ECM1 overexpression, Western blot exhibited that the expression of E-cadherin was down-regulated and Vimentin expression was up-regulated in Bel-7402 cells; the wound healing and invasion assay showed that the migration and invasion potentials of Bel-7402 cells were significantly enhanced.

**Conclusions:**

ECM1, which displayed a high expression in HCC specimens, was closely associated with clinicopathologic data and may promote migration and invasion of HCC cells by inducing EMT.

**Electronic supplementary material:**

The online version of this article (doi:10.1186/s12957-016-0952-z) contains supplementary material, which is available to authorized users.

## Background

Hepatocellular carcinoma (HCC) is the sixth most common malignancy and the third leading cause of cancer-related death in the world with about 600 thousands deaths each year [1]. Till now, curative surgical resection of HCC remains the most effective therapy. Because of high rate of metastasis and recurrence after surgery, the prognosis of HCC remains dismal with 25–39 % survival of patients in 5 years [2]. Therefore, better understandings of the molecular mechanisms that contribute to HCC invasion and metastasis are critically needed.

Some phenotypic changes and molecular events are involved in cancer cell disintegration and migration into distant organs or tissues. The epithelial-to-mesenchymal transition (EMT), which is characterized by the down-regulation or loss of epithelial markers (E-cadherin) and up-regulation of mesenchymal markers (Vimentin), is a crucial step in tumor invasion and metastasis [3–5].

Extracellular matrix protein 1 (ECM1), originally identified in 1994 as an 85 kDa glycoprotein was secreted from osteogenic mouse stromal cell line MN7 that is established from the bone marrow stroma of an adult mouse [6, 7]. Presently, a number of studies have indicated that ECM1 is involved in various biological processes, such as mineralization, cell proliferation, angiogenesis, and skin diseases [8–15]. In most cancers, ECM1 was found to promote cancer progression and invasion, and overexpression of ECM1 has been identified as an indicator of poor prognosis [10, 16–22]. Nevertheless, there are few previous studies on the role of ECM1 in HCC and EMT.

The current study is designed to evaluate the role of ECM1 expression in HCC and to elucidate the association with clinicopathological characteristics and prognosis of HCC. First, the expression of ECM1 in HCC tissues was detected using immunohistochemistry in a hospital-based cohort of patients. Next, we used the ECM1 expression plasmid transfect into Bel-7402 cells, to explore the association of ECM1 expression among E-cadherin and Vimentin.

## Methods

### Patients and tissue samples

One hundred and twenty patients with HCC who underwent curative surgical resection between 2008 and 2012 at the Provincial Hospital of Anhui Medical University were included. No patient received adjuvant therapy before surgery. These patients comprised of 93 males and 27 females, with mean age of 50.2 years (range: 21–73 years). All included patients were staged according to the sixth edition of the tumor-node-metastasis (TNM) classification of the International Union against Cancer. Tumor differentiation was defined according to the Edmondson grading system. All tissue diagnoses were confirmed by permanent histology. Seventeen samples of normal liver tissues were acquired surgically from the patients who had received an operation due to liver trauma. The institutional Ethics Committee of AnHui Provincial Hospital approval for the project was achieved before the initiation of the study. Informed consent was obtained from each patient, and the study was in compliance with the Helsinki Declaration. These patients comprised of 11 males and 6 females, with mean age of 52 years (range: 19–70 years). The clinical characteristics of these patients have no significant difference between tumor patients.

The complete follow-up data were obtained from all patients. The patients were followed up until January 1, 2015. The mean follow-up was 28.4 months (range, 3–71 months). All patients were prospectively monitored using α-fetoprotein (AFP), abdominal ultrasonography, and chest X-ray every 3–6 months after surgery. Computed tomography and/or magnetic resonance imaging were used if necessary. The overall survival (OS) was defined as the interval between the surgery and the death of patients or the last follow-up. The data were censored at the last follow-up for living patients. Disease-free survival (DFS) was measured from the date of surgery to the HCC recurrence or the last follow-up. In the DFS analysis, the data were censored for patients without tumor recurrence (Additional file 1).

### Cells and cell culture

The hepatocellular carcinoma cell line Bel-7402 was purchased from the Liver Cancer Institute of Zhongshan Hospital, Shanghai, China. The cells were routinely grown as monolayers in Dulbecco’s modified Eagle’s medium (DMEM) (Gibco BRL, New York, NY, USA) with 10 % (vol/vol) fetal bovine serum (Hyclone, Logan, UT, USA), 100 U/ml penicillin, and 100 U/ml streptomycin at 37 °C in a humidified incubator containing 5 % CO_2_.

### Immunohistochemical staining reagents

Rabbit anti-ECM1 antibody and mouse anti-Vimentin antibody were purchased from Proteintech Group Inc (USA). Horse-radish peroxidase-conjugated secondary antibody, phosphate-buffered saline (PBS), and 3,3-diaminobenzidine tetrahydrochloride (DAB) were obtained from Beijing Zhongshan Golden Bridge Biotechnology Company (China).

### Immunohistochemical staining and evaluation

Tissue specimens were promptly fixed in 10 % neutral formalin, embedded in paraffin, and cut into 3-μm sections. Immunohistochemical staining was carried out following the manufacturer’s instructions. Briefly, all sections were gradually deparaffinized with xylene and alcohol. The slides were processed with antigen retrieval by being boiled in citrate buffer (pH 6.0) for 20 min and then cooled down at room temperature. Subsequently, the slides were incubated in a 10 % hydrogen peroxide solution for 15 min to eliminate endogenous peroxidase activity. The sections were then immunostained with primary antibody of rabbit anti-ECM1 antibody (11521-1-AP, Proteintech Group, USA) and mouse anti-Vimentin antibody (ZM-0260, ZBGB-BIO) at 4 °C overnight. After rinsing with PBS for 5 min, the sections were incubated in horse-radish peroxidase (HRP)-conjugated secondary antibody (PV-6000, ZSGB-BIO) for 20 min. Being washed again, peroxidase activity was visualized using freshly prepared DAB (ZLI-9017, ZBGB-BIO) and then counterstained lightly with Harris hematoxylin. The negative controls were processed in a similar manner with PBS instead of primary antibody.

ECM1 and Vimentin were mainly located in the cytoplasm of tumor cells. The intense of immunohistochemistry staining was quantified by the percentage of positive-staining cells. The positive-staining cell <10 % was clarified as negative (−), and ≥10 % was positive (+). Ten fields were selected, and expression in 1000 tumor cells (100 cells/field) was evaluated using a high power (×400) of microscope. The immunohistochemical results were scored by three experienced pathologists, who were blinded to clinical data.

### Construction of ECM1 expression vector

Taking the cDNA of human placenta tissue as the template, RT-PCR amplification was performed and then cloned into pEGFP-N2 vector. It was sequenced to obtain the complete coding sequence of ECM1. The pEGFP-N2 plasmid (Invitrogen) was connected with the fragments of target gene after the amplification of ECM1. The double endonuclease restriction of BamH I and Xho I was performed and purified, respectively. The plasmid and fragments of target gene were recovered, which were connected with T4 DNA ligase and then cultured in LB flat plate that contained the ampicillin. The positive clone was selected and sequenced to prove the successful construction of eukaryotic expression vector pEGFP-N2-ECM1.

### Western blotting assays

Cultured cells in an exponentially growing phase and frozen tissue samples were extracted with 1 ml of lysis buffer. The protein concentration of the lysate was determined by the BCA method (KeyGen, China). The extracts containing approximately 30 μg of protein were loaded onto 10 % sodium dodecyl sulfate polyacrylamide gel electrophoresis (SDS-PAGE), electrophoresed, and then transferred to polyvinylidence difluoride (PVDF) membranes (Immobilon-P. 0.45 μm, Millipore Corp., Bedford, MA, USA). The membranes were blocked with 5 % skimmed milk in PBST (phosphate-buffered saline with 0.1 % Tween 20) for 1 h and then incubated with primary antibody of rabbit anti-ECM1 antibody (Protein Group Inc, USA), rabbit anti-Vimentin antibody (ab92574, Abcam, USA), rabbit anti-E-cadherin antibody (ab15148, Abcam, USA), and rabbit anti-β-actin (ab133626, Abcam, USA) antibody at 4 °C overnight. After being washed three times with PBST buffer, the membranes were incubated with secondary peroxidase-conjugated antibody (ZB-2308, ZSGB-BIO) for 1 h at room temperature. After being washed three more times, the protein bands were detected by enhanced chemiluminescence (Pierce, Rockford, USA) according to the manufacturer’s instruction. β-actin antibody was served as a control to confirm equal loading. Densitometry index analysis of the bands was made using gel imagery system.

### Cell migration and invasion assays

Cell migration was assayed in a transwell chamber with 24-well, 8.0-mm pore polycarbonate membrance (Corning, USA). The same membranes were precoated with matrigel (BD Biosciences, USA) for cell invasion assay. Briefly, 100 μl 1 mg/ml matrigel solution was added to the upper chamber, and then plate was immediately inverted and gelled at 37 °C for 30 min in an incubator. After 72-h transfection, cells were digested and resuspended to a concentration of 1.0 × 10^5^/ml using serum-free DMEM with 0.1 % bovine serum albumin. Two hundred-milliliter cell suspensions were seeded in the upper chamber and filled the lower chamber with 500-ml complete DMEM. The chamber was rinsed in PBS 24 h after incubation and stained with 0.1 % crystal violet for 15 min. The cells were counted using a light microscope (magnification ×200). Migrated cells were averaged from five fields per one chamber, and three chambers were used on one experiment (Additional file 2).

### Statistical analysis

All statistical analyses were performed using the statistical package SPSS 13.0 (SPSS Inc., Chicago, IL). Fisher’s exact test and the *χ*^2^ test were performed to assess associations between ECM1 expression and clinicopathological parameters. The Kaplan-Meier method was used for survival analysis, and differences in survival were estimated using the log-rank test. A multivariate survival analysis was performed for all parameters that were significant in the univariate analyses using the Cox regression model. The correlation between ECM1 and Vimentin was determined using Spearman rank correlation coefficient. The difference of ECM1 protein expression levels among cell lines was examined by using Student *t* test. A *P* value <0.05 was considered statistically significant.

## Results

### Immunohistochemical expression of ECM1 in HCC and normal liver tissues

**Fig. 1:**
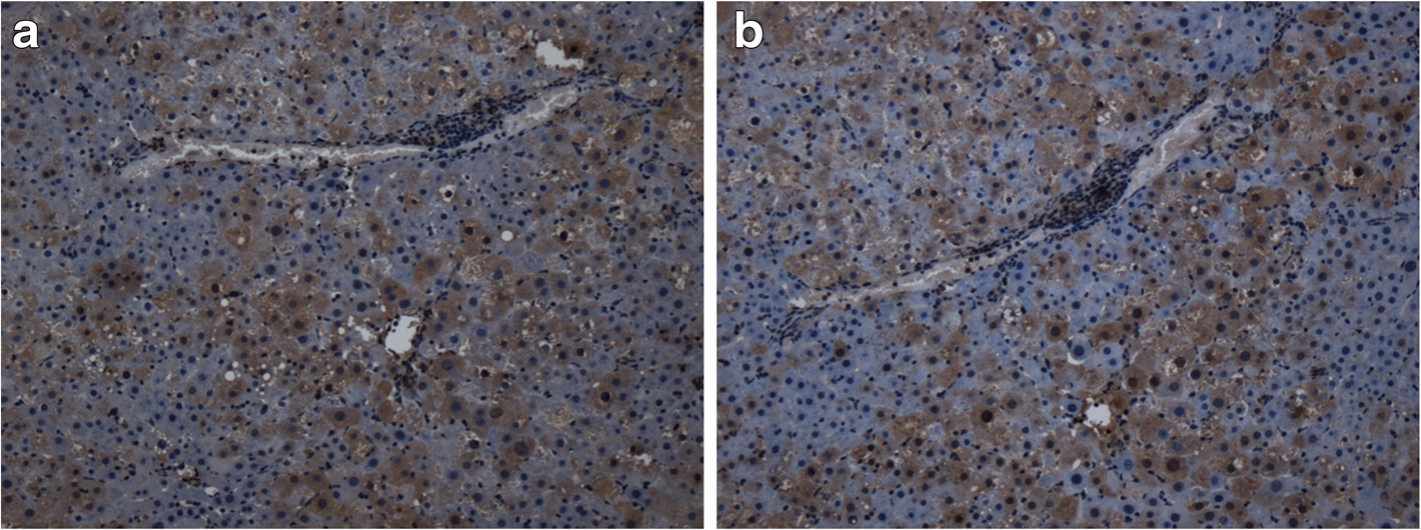
**a** Positive immunostainings for ECM1 in HCC tissues. **b** Positive immunostainings for Vimentin in HCC tissues. Both ECM1 and Vimentin mainly expressed in the cytoplasm of HCC cells. Serial sections of tumor samples were made into 3-μm sections. Original magnification at ×200

Immunohistochemical analysis revealed that both ECM1 staining was found in the cytoplasm (Fig. 1). ECM1 was detected in 73.3 % (88/120) of HCC tissues, while 23.5 % (4/17) in the normal liver tissues, respectively. The expression of ECM1 in HCC tissues was significantly higher than that in normal liver tissues (*P* < 0.01) (Table 1).

**Table 1:** Expression of ECM1 in HCC and normal liver tissues

	Cases	ECM1			
		−	+	*χ* ^*2*^	*P*
HCC	120	32	88	16.745	<0.001
Normal	17	13	4		

*P* value was generated by comparing all subgroups and analyzed by the chi-square test. *P* < 0.05 was considered as statistically significant

We evaluated the association between ECM1 expression and clinicopathological characteristics, including age, gender, tumor size, number of tumor nodules, tumor capsule, HBsAg status, serum AFP level, Edmondson’s grade, vascular invasion, Child-Pugh class, stage of liver cirrhosis, and TNM stage. ECM1 was significantly associated with TNM stage (*P* = 0.049) and venous invasion (*P* = 0.030). The expression of ECM1 was not associated with gender, age, HBsAg status, Child-Pugh class, tumor capsule, and AFP level (Table 2).

**Table 2:** Correlation between the expression of ECM1 in 120 HCC tissues and their clinicopathological characteristics

Characteristics	Case	ECM1			
		−	+	*χ* ^*2*^	*P*
Age (year)				0.001	0.978
≤50	56	15	41		
>50	64	17	47		
Gender				0.156	0.692
Male	93	24	69		
Female	27	8	19		
Nodule				0.002	0.967
Single	89	26	63		
Multiple	31	6	25		
Tumor size (cm)				0.477	0.490
≤5	65	19	46		
>5	55	13	42		
AFP (ng/ml)				0.477	0.490
≤400	55	13	42		
>400	65	19	46		
Tumor capsule				0.441	0.507
Well	66	16	50		
Poorly	54	16	38		
Edmondson’s grade				2.965	0.085
I/II	52	18	34		
III/IV	68	14	54		
HBsAg				1.108	0.293
Positive	104	26	78		
Negative	16	6	10		
Child-Pugh class				0.010	0.921
A	93	25	68		
B	27	7	20		
Vascular invasion				4.733	0.030
Absent	83	27	56		
Present	37	5	32		
Liver cirrhosis				2.472	0.291
Without/mild	67	21	46		
Moderate	38	9	29		
Severe	15	2	13		
TNM stage				6.028	0.049
I	64	23	41		
II	31	5	26		
III	25	4	21		

*P* value was generated by comparing all subgroups and analyzed by the chi-square test. *P* < 0.05 was considered as statistically significant

### Correlation with ECM1 and Vimentin expression in HCC

Vimentin is an important hallmark of EMT, and high expression of Vimentin was also observed mainly in the cytoplasmic staining. ECM1 and Vimentin expression was positive in 88 (73.3 %) and 71 (59.1 %) cases, respectively. Additionally, ECM and Vimentin were co-positive in 66 (55.0 %) cases. Using the Spearman rank test, the immunostaining expression of ECM1 was positively correlated with Vimentin expression in the HCC tissues (*r* = 0.534, *P* < 0.001) (Table 3).

**Table 3:** The correlation between ECM1 and Vimentin expression in HCC tissues

Vimentin	ECM1		*r*_s_	*P*
	Negative	Positive		
Negative	27	22	0.534	<0.001
Positive	5	66		

*P* value was generated by determined using the Spearman rank correlation coefficient. *P* < 0.05 was considered as statistically significant

### The impact of ECM1 expression on HCC prognosis

**Fig. 2:**
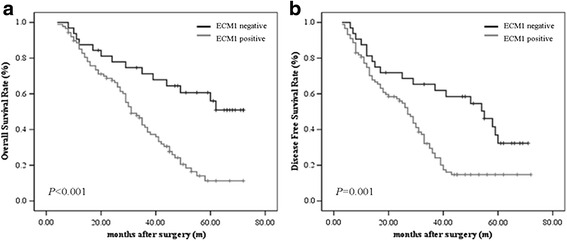
**a** Kaplan-Meier analysis of overall survival curve of HCC patients according to ECM1 expression. The HCC patients with ECM1-positive showed significantly shorter overall survival survival rates than those with ECM1-negative. **b** Kaplan-Meier analysis of disease-free survival curve of HCC patients according to ECM1 expression. The expression of ECM1 is associated with decreased disease-free survival rate

As was shown in Fig. 2, ECM1 was significantly associated with poor OS and DFS of HCC obtained by the Kaplan-Meier method using the log-rank test. In addition, the survival benefits were also found in patients with earlier TNM stage, absence of vascular invasion, single nodule, smaller tumor size, well differentiation, intact tumor capsule, and mild liver cirrhosis; in contrast, other parameters did not predict HCC prognosis (Table 4). Multivariate survival analysis enrolled above mentioned significant parameters and was performed using Cox regression. Results revealed that ECM1 expression, tumor size, number of tumor nodules, tumor capsule, vascular invasion, and liver cirrhosis were the independent prognostic factors for both OS and DFS in HCC patients after curative resection (*P* < 0.05) (Table 5).

**Table 4:** Univariate survival analysis of OS and DFS in 120 patients with HCC

Characteristics	OS			DFS		
	HR	95 % CI	*P*	HR	95 % CI	*P*
Age (>50 vs ≤50 years)	1.397	0.898–2.175	0.138	1.418	0.931–2.161	0.104
Gender (male vs female)	0.737	0.414–1.311	0.299	0.874	0.521–1.466	0.610
Nodule (multiple vs single)	3.254	2.005–5.280	<0.001	3.204	2.003–5.125	<0.001
Tumor size (>5 vs ≤5 cm)	3.747	2.374–5.913	<0.001	3.949	2.531–6.161	<0.001
Capsule (poorly vs well)	1.048	0.677–1.623	0.832	1.119	0.737–1.697	0.598
Liver cirrhosis (without/mild vs moderate/severe)	8.984	5.443–14.827	<0.001	9.798	5.955–16.119	<0.001
TNM stage (III vs I/II)	13.612	7.240–25.589	<0.001	12.074	6.480–22.500	<0.001
Edmondson’s grade (III/IV vs I/II)	1.471	0.932–2.321	0.098	1.377	0.895–2.120	0.146
Child-Pugh class (B vs A)	1.056	0.625–1.785	0.839	1.125	0.689–1.838	0.638
Vascular invasion (present vs absent)	22.957	11.683–45.112	<0.001	19.039	10.248–35.372	<0.001
AFP (>400 vs ≤400 ng/ml)	0.983	0.634–1.523	0.939	0.983	0.648–1.492	0.936
HBsAg (positive vs negative)	0.887	0.457–1.721	0.722	0.919	0.489–1.729	0.793
ECM1 (positive vs negative)	3.066	1.691–5.562	<0.001	2.294	1.360–3.872	0.002

*P* value was generated by univariate analyses using the Cox regression model. *P* < 0.05 was considered as statistically significant

**Table 5:** Multivariate survival analysis of OS and DFS in 120 patients with HCC

Characteristics	OS			DFS		
	HR	95 % CI	*P*	HR	95 % CI	*P*
Nodule (multiple vs single)	2.482	1.417–4.348	0.001	1.961	1.140–3.373	0.015
Tumor size (>5 vs ≤5 cm)	2.009	1.144–3.529	0.015	2.529	1.464–4.369	0.001
Liver cirrhosis (without/mild vs moderate/severe)	6.879	2.964–15.968	<0.001	6.703	3.078–14.598	<0.001
Vascular invasion (present vs absent)	4.291	1.848–9.960	0.001	3.430	1.563–7.527	0.002
ECM1 (positive vs negative)	4.481	2.021–9.934	<0.001	3.289	1.601–6.758	0.001

*P* value was generated by multivariate survival analysis performed for all parameters that were significant in the univariate analyses using the Cox regression model. *P* < 0.05 was considered as statistically significant

### Effect of ECM1 on expression of E-cadherin and Vimentin protein in HCC cells

**Fig. 3:**
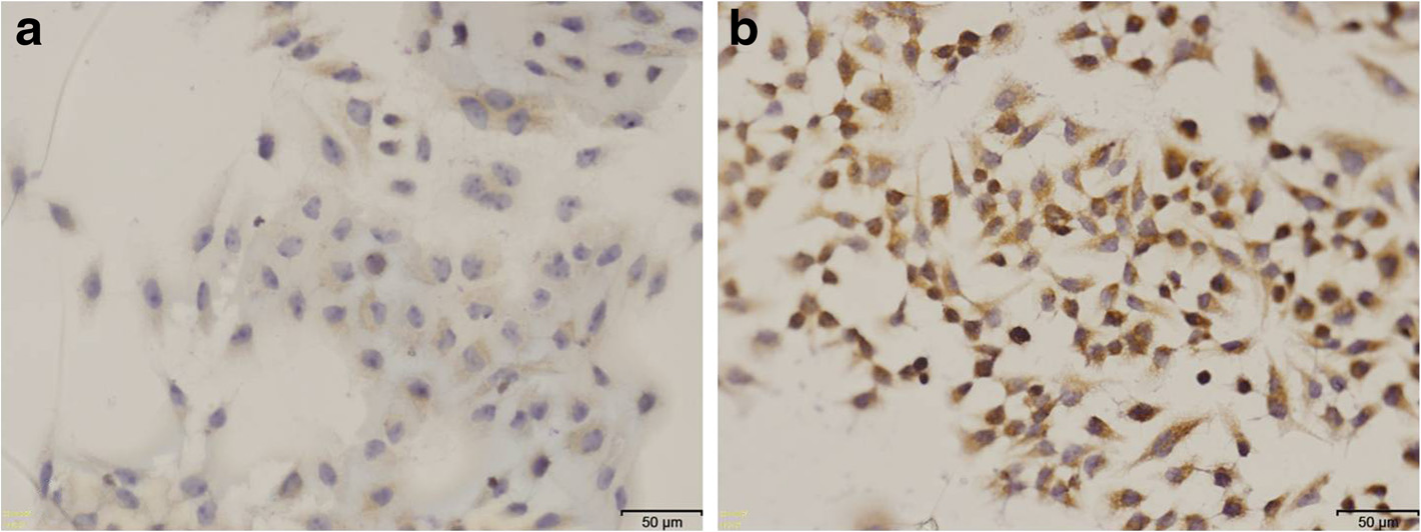
**a** Representative pictures of ECM1 staining in Bel-7402 cells, ECM1 mainly expressed in the cytoplasm of cells. **b** Representative pictures of ECM1 staining in ECM1-Bel-7402 cells. The ECM1 level of Bel-7402 cells was significantly increased detected by immunohistochemical staining. Original magnification at ×200

**Fig. 4:**
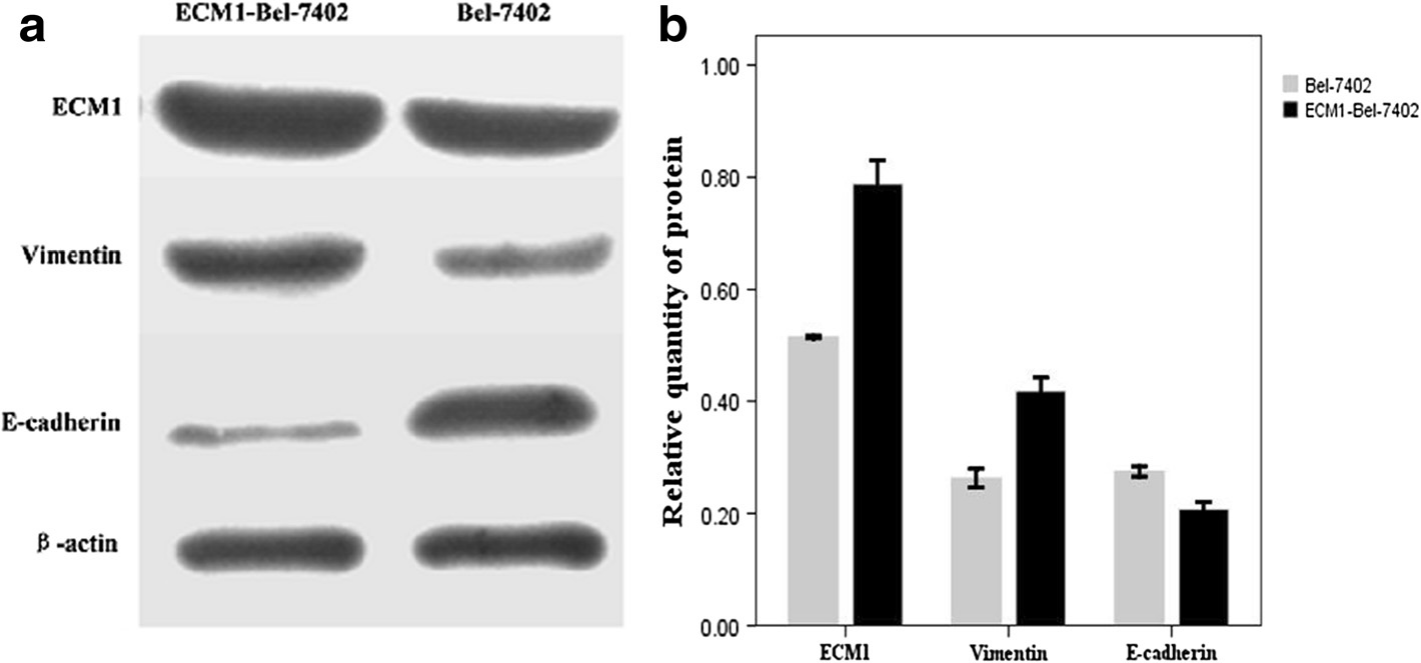
**a** Changes of ECM1 and EMT-related protein (E-cadherin and Vimentin) expression in Bel-7402 cells were detected by Western blot after ECM1 transfection. The increased expression of ECM1 could lead to the up-regulated expression of Vimentin protein and down-regulated expression of E-cadherin protein in Bel-7402 cells. **b** The relative quantity of three protein expressed levels in cells with significant difference (ECM1: *t* = 12.909, *P* < 0.01; Vimentin; *t* = 10.564, *P* < 0.01; E-cadherin: *t* = −8.558, *P* = 0.002). Relative quantity of three protein expressed levels in cells. Data are mean ± SD of three replicates. (*P* < 0.05)

The ECM1 level of Bel-7402 cells was significantly increased detected by immunohistochemical staining (Fig. 3). As shown in Fig. 4, the increased expression of ECM1 could lead to the up-regulated expression of Vimentin protein and down-regulated expression of E-cadherin protein in Bel-7402 cells. The relative quantity of three protein expressed levels in cells with significant difference (ECM1: *t* = 12.909, *P* < 0.01; Vimentin; *t* = 10.564, *P* < 0.01; E-cadherin:*t* = −8.558, *P* = 0.002).

### ECM1 overexpression induces cell migration and invasion

**Fig. 5:**
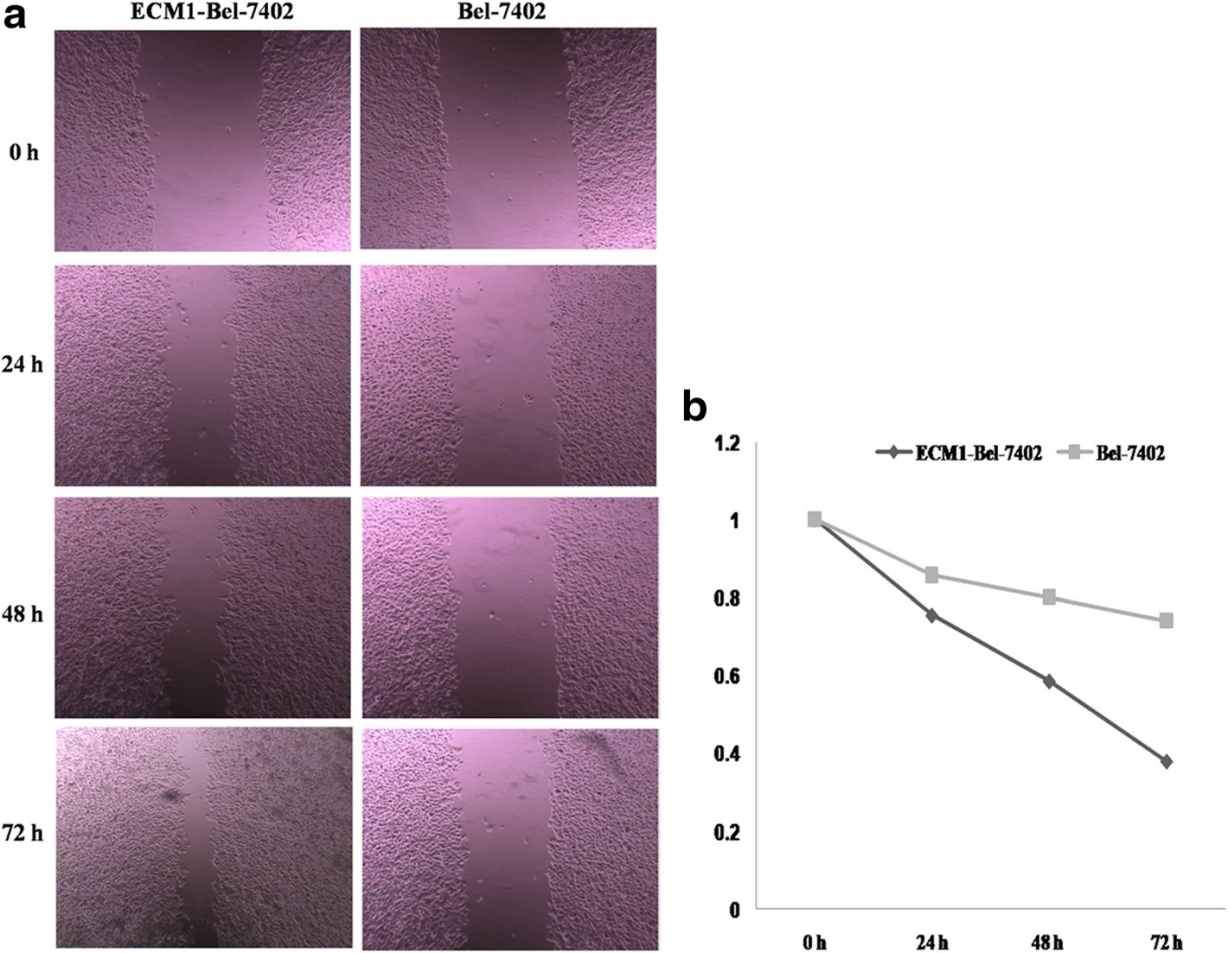
**a** Effect of ECM1 on the migration capacity of Bel-7402 cell by the wound healing assay at 24, 48, and 72 h (×40). **b** Statistics of analysis of migration rate date in Bel-7402 and ECM1-Bel-7402 cells. The wound healing assay revealed that the migration rates of the cells in the Bel-7402 group infected with pEGFP-N2-ECM1 were significantly increased compared with those without transfected group (*F* = 110.592, *P* < 0.01). Data are mean ± SD of three replicates. (*P* < 0.05)

**Fig. 6:**
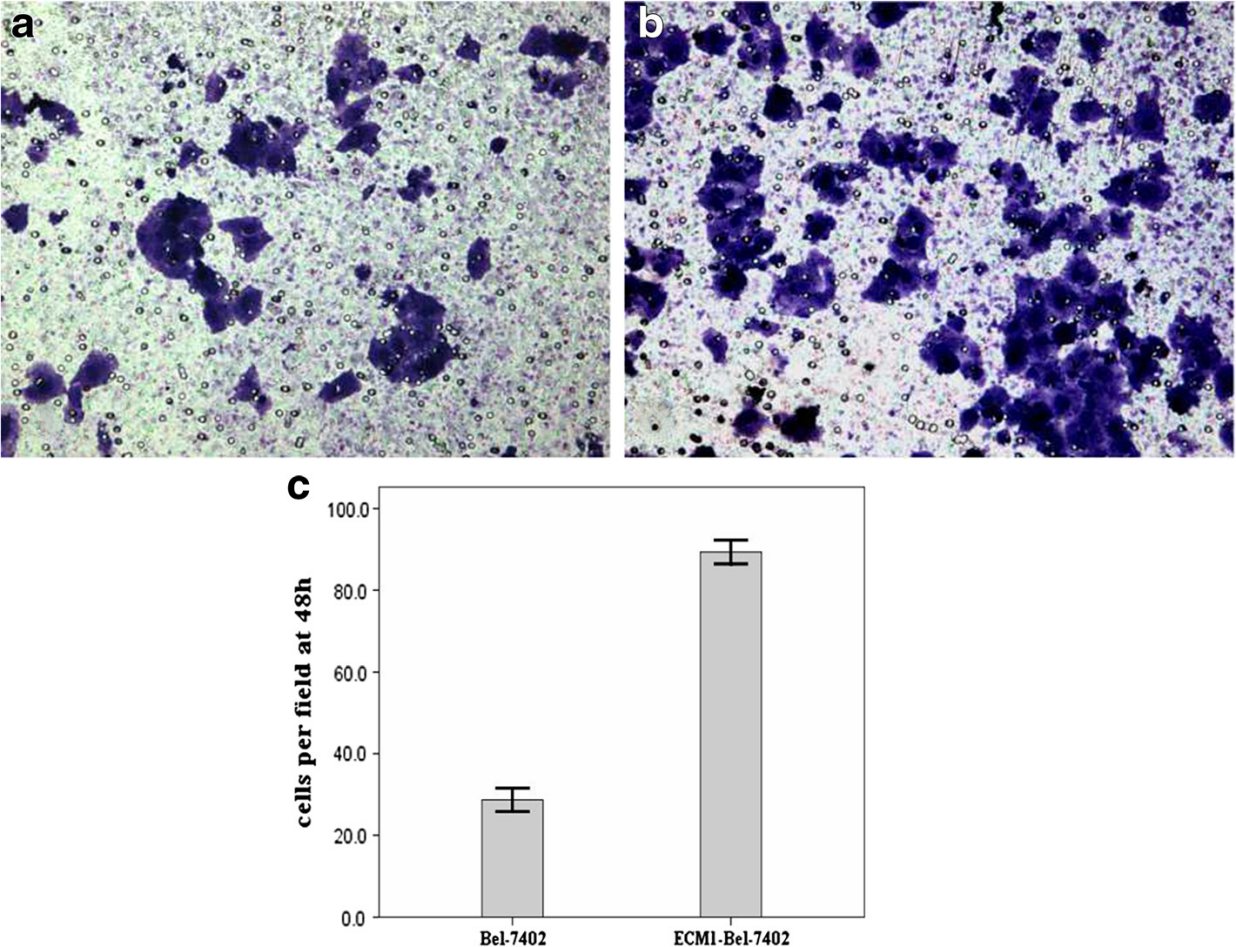
**a**, **b** Effect of ECM1 on the invasion capacity of Bel-7402 and ECM1-Bel-7402 cells by invasion assay (×200). **c** Statistics of analysis of cells per field in Bel-7402 and ECM1-Bel-7402 cells at 48 h. Compared with the control group, after the up-regulated expression of ECM1, the number of invading Bel-7402 cells was significantly increased (89.3 ± 2.5 cells/well vs 28.7 ± 2.5 cells/well) (*t* = 29.524, *P* < 0.01). Data are mean ± SD of three replicates (*P* < 0.05)

To evaluate the role of ECM1 in the regulation of HCC cells migration, we carried out a wound healing assay at 24 h post-infection. The wound healing assay revealed that the migration rates of the cells in the Bel-7402 group infected with pEGFP-N2-ECM1 were significantly increased compared with those without transfected group (*F* = 110.592, *P* < 0.01 (Fig. 5). Subsequently, we assessed the effect of ECM1 on cell invasion with a transwell assay. As shown in Fig. 6, compared with the control group, after the up-regulated expression of ECM1, the number of invading Bel-7402 cells was significantly increased (89.3 ± 2.5 cells/well vs 28.7 ± 2.5 cells/well). (*t* = 29.524, *P* < 0.01).

## Discussion

Cytoplasmic ECM1 expression seems preferentially expressed in metastatic epithelial tumors, and it has been observed in breast cancer, thyroid carcinoma, laryngeal carcinoma, etc. [17, 18, 20, 21]. To date, little is known about the association between ECM1 expression and clinicopathological characteristics and prognosis of HCC. In the present study, HCC patients with positive ECM1 expression had significantly poor survival rates, especially in the subgroups of patients with invasive phenotypes, such as those with advanced TNM stage and vascular invasion. Using Cox multivariate regression analysis, ECM1 expression was an independent prognostic factor for OS and DFS of HCC patients. Therefore, it was proposed that the presence of ECM1 would be efficient for the prediction of HCC metastasis and prognosis.

Although many studies have reported that ECM1 affected not only the malignant cellular proliferation, but the cancer cell migration and invasion, the exact mechanisms by which ECM1 promoted tumor progression remained unclear. HCC is one of the most common malignant tumors in China, but few patients have chance to radical excision, postoperative recurrence rate is extremely high, and migration and invasion are the main factors of recurrence of HCC. In the migration of tumor involving a variety of regulatory mechanism, EMT is one of the important mechanisms, which was first discovered at key transition steps during embryogenesis and was the critical event that mediated tumor metastasis [23]. EMT refers to the epithelial cells in certain cases to the phenomenon of mesenchymal cells; the main features for the loss of epithelial phenotype and the get of mesenchymal phenotype result in heightened cell motility and invasiveness through diminished cell–cell and cell–matrix adhesion, reorganization of the cytoskeleton, and remodeling of the ECM [24–26]. Vimentin has been recognized as a very important marker for EMT, and its overexpression has been strongly associated with metastatic phenotype and poor prognosis. More and more evidence suggest that EMT plays an extremely important role during the process of the spread of cancer of HCC [27, 28].

Primary studies have reported that ECM1 may promote EMT progression and increases the CSS phenotypes through the stabilization of β-catenin protein by MUC1 [29]. But whether ECM1 can promote migration of HCC through the induction of EMT is unclear. This experiment which mainly studies ECM1 in the role of EMT-induced liver cancer metastasis further clarifies the important effect of the migration in liver cancer invasion. Our previous study suggests that ECM1 is over-expressed in both HCC cells and tissues compared with their benign counterparts [19]. On this basis, this research transfects ECM1 into Bel-7402 HCC cell line, induced by exogenous ECM1 expression. In this study, we found that the up-regulated expression of ECM1 was able to induce EMT. The expression of E-cadherin significantly declined, and Vimentin was significantly increased, which are very important markers for EMT, suggesting that ECM1-Bel-7402 cells can be induced to the occurrence of EMT. The tumor progression and metastasis-inducing capability in vitro of HCC cell line were also significantly enhanced, which is consistent with previous findings in this study.

## Conclusions

Comprehensive above research results, our study indicated that the expression of ECM1 in HCC tissues was significantly associated with invasive phenotypes of HCC. ECM1 is a novel diagnostic marker for predicting the prognosis of HCC patients, although further study with a larger sample size was needed to confirm the current findings. As a secretory glycoprotein, detection of serum ECM1 level in HCC patients should be considered. We also found that ECM1 could induce liver cancer cell migration by EMT, this may provide a new train of thought for HCC effective diagnosis and treatment. But the intrinsic mechanism of ECM1 promoting the migration of tumor is very complex, and the induction of EMT may only be one of these links; therefore, other mechanisms of ECM1 by which in the process of HCC cell migration still need to be further discussed.

## Abbreviations

AFP, α-fetoprotein; DAB, 3,3-diaminobenzidine tetrahydrochloride; DFS, disease-free survival; DMEM, Dulbecco’s modified Eagle’s medium; ECM1, extracellular matrix protein 1; EMT, epithelia-mesenchymal transition; HbsAg, hepatitis B surface antigen; HCC, hepatocellular carcinoma; OS, overall survival; PBS, phosphate-buffered saline; TNM, tumor-node-metastasis

## Additional files

Additional file 1: The changes of the expression of ECM1 in Bel-7402 cells effect some protenin levels, and the ability of migration and invasive potential of Bel-7402 cells. (XLSX 40 kb) Additional file 2: One hundred twenty patient’s clinical characteristics and the complete follow-up data. (XLS 36 kb)
